# The Circular Life of Human CD38: From Basic Science to Clinics and Back

**DOI:** 10.3390/molecules25204844

**Published:** 2020-10-21

**Authors:** Alberto L. Horenstein, Angelo C. Faini, Fabio Morandi, Cristiano Bracci, Francesco Lanza, Nicola Giuliani, Aneel Paulus, Fabio Malavasi

**Affiliations:** 1 Dipartimento Scienze Mediche, Università di Torino, Centro Ricerche Medicina Sperimentale (CeRMS) and Fondazione Ricerca Molinette Onlus, 10121 Torino, Italy; horenstein.al@gmail.com (A.L.H.); angelo.faini@edu.unito.it (A.C.F.); cristiano.bracci@edu.unito.it (C.B.); 2 U.O.C. Laboratorio Cellule Staminali Post Natali e Terapie Cellulari, IRCCS Istituto Giannina Gaslini, 16121 Genoa, Italy; fabiomorandi@gaslini.org; 3 Section of Hematology, Hospital of Ravenna, 48121 Ravenna, Italy; francesco.lanza@auslromagna.it; 4 Laboratorio di Ematologia, Dipartimento di Medicina e Chirurgia, Università di Parma, 43121 Parma, Italy; nicola.giuliani@unipr.it; 5 Department of Cancer Biology Mayo Clinic, Jacksonville, FL 32224, USA; Paulus.Aneel@mayo.edu; 6 Division of Hematology & Oncology, Mayo Clinic, Jacksonville, FL 32224, USA

**Keywords:** CD38, multiple myeloma, therapeutic antibodies

## Abstract

Monoclonal antibodies (mAbs) were initially considered as a possible “magic bullet” for in vivo elimination of tumor cells. mAbs represented the first step: however, as they were murine in nature (the earliest experience on the field), they were considered unfit for human applications. This prompted the development of techniques for cloning the variable regions of conventional murine antibodies, genetically mounted on human IgG. The last step in this years-long process was the design for the preparation of fully human reagents. The choice of the target molecule was also problematic, since cancer-specific targets are quite limited in number. To overcome this obstacle in the planning phases of antibody-mediated therapy, attention was focused on a set of normal molecules, whose quantitative distribution may balance a tissue-dependent generalized expression. The results and clinical success obtained with anti-CD20 mAbs revived interest in this type of strategy. Using multiple myeloma (MM) as a tumor model was challenging first of all because the plasma cells and their neoplastic counterpart eluded the efforts of the Workshop on Differentiation Antigens to find a target molecule exclusively expressed by these cells. For this reason, attention was turned to surface molecules which fulfill the requisites of being reasonably good targets, even if not specifically restricted to tumor cells. In 2009, we proposed CD38 as a MM target in virtue of its expression: it is absent on early hematological progenitors, has variable but generalized limited expression by normal cells, but is extremely high in plasma cells and in myeloma. Further, regulation of its expression appeared to be dependent on a variety of factors, including exposure to all-trans retinoic acid (ATRA), a potent and highly specific inducer of CD38 expression in human promyelocytic leukemia cells that are now approved for in vivo use. This review discusses the history of human CD38, from its initial characterization to its targeting in antibody-mediated therapy of human myeloma.

## 1. Premise

The seminal paper by Köhler and Milstein [1] ended with the suggestion that monoclonal antibodies (mAbs) may act as “magic bullets” for in vivo elimination of tumor cells. The suggestion was appealing in virtue of its internal linearity: One identifies the proper target molecule and then raises specific antibodies.

While antibodies represented the first step, these were mainly murine-based reagents (the initial experience in the field) and were considered unfit for human applications. This prompted the development of techniques for cloning the variable regions of conventional murine antibodies, genetically mounted on human IgG. The last step of the process (which indeed took years) was the design of the construction of fully human reagents. Multiple other approaches are now available [2]. 

The choice of the target molecule is also problematic: in a tumor, bona fide cancer-specific targets are quite limited in number. To overcome this issue and when planning antibody-mediated therapy, attention was prioritized to a set of normal molecules, whose quantitative distribution may balance a tissue-dependent generalized expression. The experience and the clinical success obtained with anti-CD20 mAbs revitalized the interest in this strategy [3]. Similar strategies were applied for therapeutic targeting of CD22 [4]. Utilizing multiple myeloma (MM) as a tumor model, a first limitation is represented by the fact that plasma cells and their neoplastic counterpart eluded the efforts of the Workshop on Differentiation Antigens to find a target molecule exclusively expressed by these cells. For this reason, attention was focused on surface molecules, which answer the requisites of being reasonably good targets, even if not strictly restricted to tumor cells specifically. The list of such candidate molecules for MM is long and summarized elsewhere [5].

In 2009, we proposed CD38 as an MM target in virtue of its expression: Absent on early hematological progenitors, variable but generalized limited expression by normal cells, but extremely high in plasma cells and on their tumor counterpart MM [6].

Further, the regulation of its expression appeared to be dependent on various factors. A candidate for increasing surface expression was identified by analyzing models of cell-differentiation therapy. We reported that all-trans retinoic acid (ATRA) is a potent and highly specific inducer of CD38 expression in human promyelocytic leukemia cells. We also observed that ATRA-induced expression of CD38 in myeloid cells is mediated through the retinoic acid α receptor (RARα). Increased CD38 protein antigen levels were also observed in normal CD34^+^ bone marrow exposed to ATRA, but not on normal circulating granulocytes. These effects are independent of differentiation and mediated by RARα. ATRA is now approved for in vivo use [7,8,9].

Our experience on CD38 biology, as studied in in vitro and in vivo (animal models) experiments, has been steadily accumulating along with potential benefits in translation to clinical medicine.

A brief overview on the main characteristics of the molecule is necessary to track the evolution of the molecule in medical practice.

## 2. Gene and Structure

The human CD38 gene is an eight-exon complex located on chromosome 4p15 which, with its duplicate CD157, forms part of the eukaryotic nicotinamide adenine dinucleotide (NAD^+^) glycohydrolase/ADP-ribosyl cyclase gene family [10]. The restriction endonuclease pvu identifies a bi-allelic polymorphism for the gene. The frequency in the Caucasian population is 14% and 86%, respectively [6]. In conjunction with the polymorphic site, there is a 900 base pair CPG island with two potential SP1 binding sites. The CPG island plays a role in the regulation of CD38 expression. 

**Figure 1:**
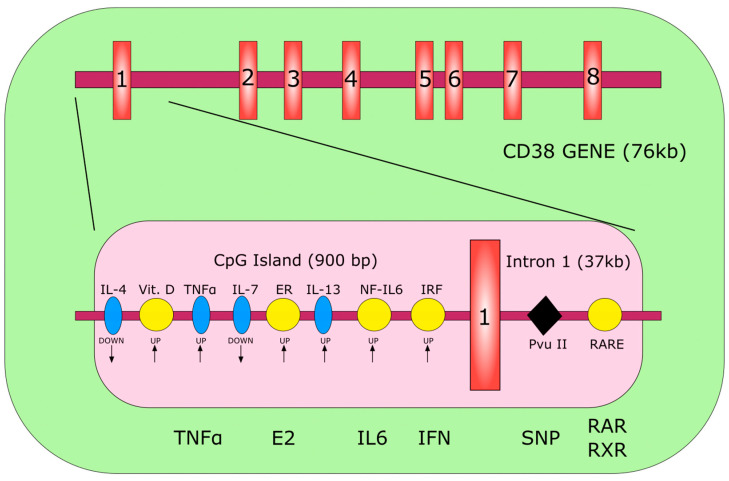
Schematic representation of the human CD38 gene. The red rectangles identify the single exons. The pink rectangle depicts the CpG island. The yellow circles indicate specific responsive elements in the regulatory region of the gene. The blue ellipses indicate regulatory interleukins for which no responsive element has been identified. The black diamond highlights the polymorphic site. ER, estrogen-responsive element; IRF, interferon-responsive element; RARE, retinoic acid-responsive element; E2, estrogen; SNP, single nucleotide polymorphism; RAR, retinoic acid receptor. Modified from [6].

The organization of the gene is described in details [6,9]. Schematic organization is reported in Figure 1.

CD38 was first identified in the late 1970s during the pioneering project of the Workshop on Human Differentiation Antigens using murine monoclonal antibodies (mAbs) as analytical tools. First observed on thymocytes and T lymphocytes and known as the “T10 activation molecule”, CD38 is now confirmed as expressed at variable levels both within and outside the immune system [11].

Biochemically, CD38 is a 43.7 kDa molecule, as measured by direct β-imaging analysis of the metabolic labeled type II transmembrane glycoprotein [12,13,14].

Specific ligation of the molecule by murine anti-CD38 mAbs causes down-modulation of the surface molecule, yielding a 39-kDa soluble protein (sCD38) with identical characteristics to the native antigen and secreted in normal and pathological fluids [15,16].

The existence of a soluble counterpart sharing immunological features with the membrane paved the way to the identification of a ligand of human CD38. Initially termed with the name of the original Moon-1 hybridoma, the molecule was later clusterized as CD31/PECAM-1 [17]. In the U937 myeloid line, circulating CD38 was found to maintain its ability to bind CD31, which is a 120-kDa cell surface ligand present on endothelial cells [18,19].

## 3. Gene Regulation

The CD38 promoter is sensitive to vitamins, hormones, cytokines, different retinoids and to the non-selective histone deacetylase inhibitor panabinostat. The action of the latter is apparently restricted to MM cells.

DNA methyltransferases (DNMTs) methylate cytosine residues in cpG islands and remodel chromatin. Azacytine (AZA) and decitabine (DEC), recently approved for in vivo treatment, cause passive loss of cytosine methylation in daughter cells. It was shown that AZA and DEC increase CD38 expression, through direct and indirect mechanisms. One of these is represented by the secretion of TNFα. 

A different mechanism of CD38 regulation was also recently reported, which operates through the action of the tumor suppressor miR-26a [20].

## 4. CD38 Functions

Different functions have been attributed to the molecule:

### 4.1. As a Receptor

Distinct approaches indicated that CD38 is involved in complex and apparently unrelated processes, such as cell activation and proliferation, egg fertilization, muscle contraction, hormone secretion, and immune responses. However, the fact that T10 was the tenth molecule identified in T lymphocytes profoundly influenced the orientation of the successive investigations on CD38 towards an immunological direction. Most of the above functional effects might be interpreted as secondary to an interplay between CD38 and an unknown ligand (see previous section). Anti-CD38 agonistic mAbs were utilized to mimic the effects of a putative ligand. This approach arose from the observation that a limited number of the different anti-CD38 mAbs available at the time were agonistic, i.e. their ligation of the target molecule was followed by delivery of positive signals. The results obtained led the conclusion that the molecule may act as receptor, as further strengthened by the results of co-modulation experiments, indicating that CD38 displays lateral associations and likely shares signaling pathways [21].

This observation suggested that CD38 is per se inept to act as a receptor (due to a very short cytoplasmic tail), but could overcome this limitation by parasitizing professional receptors. This was confirmed on T lymphocytes [22], on B lymphocytes [23], on NK cells [24,25] and on monocytes [26].

### 4.2. As an Ectoenzyme

An unexpected observation came to complete the previous findings, which attributed to CD38 an immunological role. Indeed, H. C. Lee, a biochemist working in unrelated fields, came across CD38 while studying new intracellular messengers in the sea mollusk *Aplysia*, pre-dating Homo sapiens by 900 million years. During the course of his work, he made the surprising finding on the striking similarity between human CD38 and the enzyme ADP ribosyl cyclase, purified from *Aplysia*. CD38 was then confirmed to be a key enzyme metabolizer of nicotinamide adenine dinucleotide (NAD^+^). Acting as a metabolic sensor in both normal and tumor cells, at physiological pH CD38 catalyzes the transformation of extracellular β-NAD^+^ (NAD^+^-glycohydrolase activity) to produce ~2% of Cyclic ADP Ribose (cADPR) (cycling activity) and ~90% of ADPR (hydrolytic activity), simultaneously releasing nicotinamide (Nam) [27]. At intra- and extra-cellular levels and at acidic pH in presence of the co-substrate nicotinic acid (NA), CD38 also catalyzes the metabolization of a phosphorylated form of NAD^+^ (NADP^+^) to produce nicotinic acid adenine dinucleotide phosphate (NAADP) (NADP^+^-ase activity) and ADPRP (hydrolytic activity). All final products (cADPR, ADPR and NAADP) are regulators of intracellular ionic calcium signaling [6]. The resulting implication is that NAD^+^ is a physiological substrate ligand of CD38, a nucleotide-metabolizing ectoenzyme which is also part of a set of molecules involved in the catabolism and scavenging of extracellular nucleotides. Indeed, NAD^+^, as is also the case for adenosine triphosphate (ATP), is increased in the extracellular space during inflammatory or neoplastic conditions, where these adenine nucleotides are believed to act as danger signals, alerting the immune system to avoid possible tissue damages through binding to P2-purinergic receptors [28]. In order to restore the extracellular homeostasis, a scavenging circuit is operated by nucleotide-catabolizing ectoenzymes that generate nucleosides (e.g., adenosine (ADO) and inosine (INO)), which can re-enter the cell, to reconstitute the pool of nucleotides. This process also results in the synthesis of compounds that play a critical role in cell homeostasis and metabolism, beyond that of nucleotide recycling. It was initially thought that one characteristic shared by several nucleotide-metabolizing ectoenzymes was their ability to function in environments containing only trace amounts of the substrate and where the final product would be used prevalently inside the cell. This initial view was later refined, confirming that substrates and final products are not so topologically confined [29,30].

### 4.3. CD38 Connections

Findings and evidence coming from unrelated fields, fueled the interest in the CD38 molecule and opened the way for new avenues of research. For instance, the availability of knock out (KO) animal models made it possible to study, in vivo, the effects attributable to the absence of the molecule. KO mice displayed impaired immune response in respiratory districts [31], and other problems. However, genetic ablation of CD38 in mice was noted to be insufficient for interfering with relatively normal development, reproduction and parental care.

These studies widened the scientific approaches to CD38 and diversified its applications. The most striking results came from neurophysiology, where CD38 emerged as a regulator of oxytocin release [32]. Oxytocin is a hormone apparently more related to behavior than immunity. Another unexpected field was the role of NAD^+^ in the control of aging [33].

However, we maintained our initial belief that the best suggestions about the role of an orphan molecule, such as CD38, would come from human diseases, where it may play a role in pathogenesis or act as a simple marker [34]. Moreover, human diseases as models have become extremely informative following the introduction of therapeutic antibodies. Indeed, and from a basic science perspective, a patient with a known disease, such as MM, and treated with anti-CD38 antibodies represents a modality of analysis superior to all in vitro models. Speculatively, information coming from patients affected from a disease taken as a model represents a source of knowledge likely able to improve the potential of antibody therapy.

This review will be limited to the analysis of a set of information derived from the involvement of CD38 in distinct human pathologies and the increase in basic knowledge derived from antibody-mediated therapy in human MM. 

### 4.4. Cytolytic and Catalytic CD38-Dependent Effects of Therapeutic mAbs in MM

Some of the hypotheses raised during the in vitro studies of the CD38 molecule were confirmed by the results collected during the in vivo application of therapeutic antibodies. The picture was made easier by the availability of a panel of therapeutic antibodies approved for in vivo use.

**Figure 2:**
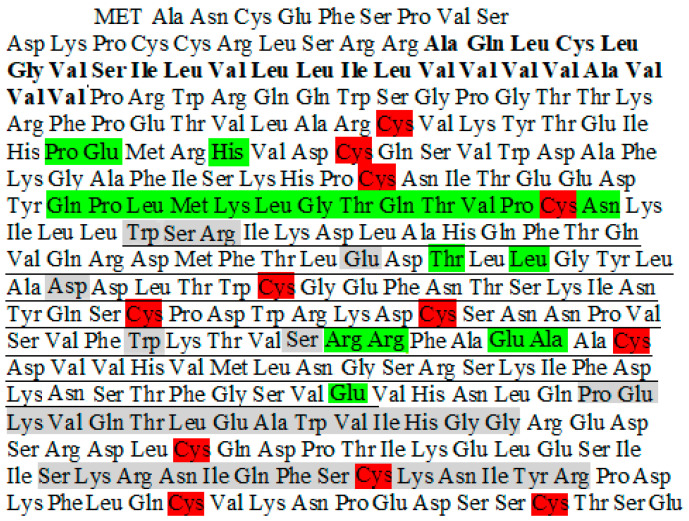
Comparison of Isatuximab (ISA) and Daratumumab (DARA) epitopes on human CD38. The gray shading denotes the epitope of CD38 for DARA, the green shading denotes the epitope of CD38 for ISA. The red shading denotes the cysteines (Cys) forming disulfide bonds necessary for maintaining the 3D-structure of the CD38 molecule. The catalytic extracellular domain of CD38 span over 100 residues in the CD38 sequence, from Trp125 to Glu226 (underlined). The intra-membrane CD38 sequence is in bold.

The first human antibody to be approved for monotherapy is Daratumumab (DARA for short, Darzalex, Janssen), followed by Isatuximab (ISA for short, Sarclisa, Sanofi) used in combination with other anti-MM therapies. MOR202 (Morphosys) and TAK-079 (Takeda) are other candidates in advanced stages of development. A panel of other reagents (humoral and cell-based) are in preparation [35]. The results from clinical practice are indeed encouraging, wherein anti-CD38 mAbs show extension of overall survival and quality of life in patients with relapsed MM. On the other side, MM provided a model in which to confirm hints coming from basic science, likely useful to improve the efficiency of antibody therapy [36]. The human CD38 epitopes recognized by ISA and DARA (Figure 2) have been identified based on crystal structure studies [14,37]. DARA, a human mAb, reacts with the CD38 molecule in epitopes distant from its catalytic site. ISA, a humanized mAb, displays peculiar characteristics recently described [38,39]. The effects of ISA are reported as being extremely sensitive to the number of CD38 molecules present per cell surface. Indeed, a threshold limit was identified, beyond which the antibody does not feature its functional activity. This peculiar behavior should be considered a beneficial property of the antibody, through which normal cells and immune effector cells (which typically have low CD38 density) are left unaltered. ISA is reported as reacting with an epitope, which encompasses (at least in part) the catalytic site of the CD38 ectoenzyme. As shown in Figure 2, ISA binds to a specific 23-amino acid discontinuous epitope, which includes a continuous sequence of 12 amino acids on the CD38 protein. DARA binds to two continuous sequences of 15 and 14 amino acids each, all of them located outside the catalytic site of CD38 (Figure 2).

Under normal conditions, CD38 is expressed at relatively low levels on myeloid and lymphoid cells and in some non-hematopoietic tissues. In contrast, normal plasma cells and MM malignant plasma cells have high levels of CD38 expression, making CD38 an attractive target for therapeutic mAbs to treat MM. 

DARA confirmed that the expected cytolytic effects mediated by the therapeutic IgG were sided by signals also exerted on positive (T and B lymphocytes) as well as negative effectors (monocytes and myeloid-derived cell suppressor cells). What remains a matter of debate, are the effects of DARA exerted on NK cells, which decrease rapidly after injection and recover in number months after therapy cessation [40].

CD38 as a NAD^+^-glycohydrolase ectoenzyme catalyzes, under physiological conditions, the metabolism of NAD^+^ to ADPR and NAM. When pathological conditions (such as, inflammation or neoplastic growth) induce acidification of the environment, CD38 metabolizes NADP^+^ to NAADP, using NA. Upon binding by therapeutic mAbs, a significant conformational change was observed in CD38 [41]. However, the overall configuration of key residues involved in the CD38 enzymatic activity is maintained and the catalytic site remains accessible to the substrate. In fact, the NAD^+^-glycohydrolase enzymatic activity of CD38 was not modified in presence of any anti-CD38 human therapeutic mAb (or murine mAb) as measured by HPLC assays (Horenstein et al., 2019 unpublished). These data support the extended evidence that CD38 has to be considered just an NAD^+^-glycohydrolase [42], whose activity is barely modified by any known murine or human mAb.

As shown previously, CD38 is also endowed with both cyclization and hydrolytic functionalities. Notably, both therapeutic mAbs inhibit with different potency cADPR formation (cyclase function) by an allosteric antagonism and increase ADPR formation (hydrolase function) by an agonist mechanism, respectively. These CD38 enzymatic activities were experimentally analyzed by our group, in the presence of anti-CD38 mAbs using CD38-expressing cells (e.g., MM plasma cells obtained from bone marrow biopsies as well cultured MM cell lines). Accordingly, cells were incubated with: NAD^+^ (for cyclase activity) and cADPR (for hydrolase activity) as substrates in the absence or presence of ISA, DARA, and control antibodies. DARA and ISA inhibited the enzymatic cyclase function of CD38+ cells and cell lines (range: 25–45%). Both mAbs were shown, however, to activate (range: 15–25%) the hydrolytic activity of CD38-expressing cells when incubated with cADPR a gift from H.C. Lee as the substrate [43]. These findings confirmed previous reported data [35,44]. 

Surprisingly, ISA was recently reported to inhibit the hydrolase activity of CD38, even if the experimental conditions were not described [35].

Consequently, future studies on the modulation of CD38 enzymatic activities by therapeutic mAbs should deal with the analysis of physiological downstream effects, such as insulin release [45], or adenosine production [43], to know if modulation of the cyclase/hydrolase functional activities alters the distal effect of CD38 signals [6]. More importantly, the influence of DARA and ISA on the enzymatic activities of CD38 have not yet been fully evaluated in vivo.

## 5. CD38-Controlled Activities and Metabolic Adaptation during Diseases

**Figure 3:**
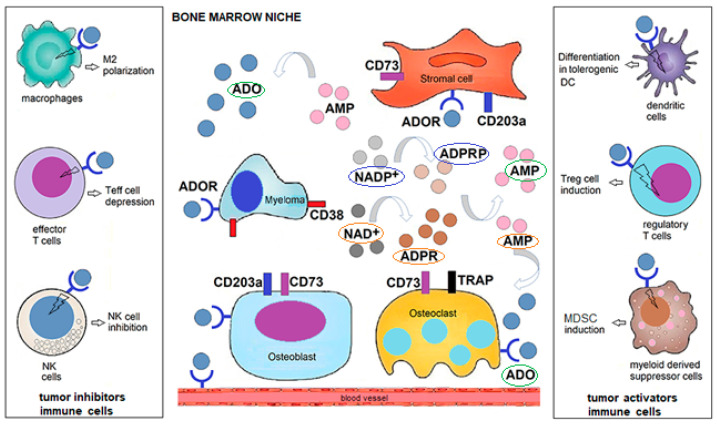
Human malignant multiple myeloma (MM) cells under hypoxic and acidic conditions support the production of adenosine (ADO) inside the BM niche for the generation of an anergic site. ADO is obtained from NAD^+^ or NADP^+^ which are metabolized based on pH status by a (multicellular) cascade of ectonucleotidases: CD38/CD203a/CD73 at pH 6–7.5 or CD38/CD203a/TRAP at pH 4.5–6.5, respectively. Once NAD^+^/NADP^+^ are disassembled into byproducts: cADPR/ADPR/AMP/ADO or NAADP/ADPRP/AMP/ADO, respectively, the immunosuppressive nucleoside ADO is accumulated in variable amounts in the BM niche. Most of ADO is taken up by specific type 1 ADORs expressed by malignant plasma cells, bone cells or immune cells inside the BM niche that can either block T cytotoxic (T_eff_) cells and NK cells able of destroying malignant cells. A second key effect is the increase in the number of regulatory T-cells (T_regs_) and mesenchymal-derived stromal cells (MDSC), among others, which prevent immune cells from responding to the tumor. Abbreviations: NAD^+^, nicotinamide adenine dinucleotide; ADPR, adenosine diphosphate ribose; cADPR, cyclic adenosine diphosphate ribose; NAADP, Nicotinic acid adenine dinucleotide phosphate; ADO, adenosine; ADOR, P1 ADO receptor.

CD38 is highly and uniformly expressed by malignant plasma cells [46] and such a feature has significant ramifications on: (1) the enzymatic reaction of CD38 within the tumor microenvironment (e.g., Bone marrow [BM] niche); (2) the type and degree of expression of CD38-associated ectonucleotidases (e.g., CD39, CD203a, CD73, TRAP, CD26), and the triggering of a multicellular (e.g., the type of immune and non-immune cells present in the BM niche) pathway for adenosine (ADO) production; and (3) their dependency on the environmental metabolic conditions (e.g., pH, oxygen levels) inside the BM niche (Figure 3).

These features of MM as a disease model are relevant to understand how the intracellular conditions of MM cells and the BM environment influence CD38. To address this, it is necessary to identify the metabolic conditions in which MM cells grow. Normal cells employ mitochondrial oxidative phosphorylation (OXPHOS) to generate energy, whereas malignant plasma cells need a higher supply of energy. Therefore, MM cells exploit different mechanisms, among which they salvage glycolytic metabolism for energy production and scavenging of nucleotides and other macromolecules. This metabolic shift results in the secretion of most of the glucose-derived carbon as lactic acid, which generates an acidic tumor microenvironment and is likely advantageous for malignant plasma cell survival in the BM niche. Furthermore, a pathologic extracellular environment is also generally hypoxic as a consequence of malignant cell proliferation, altered vascularization and metabolic shift. In such an acidic hypoxic niche, lactic acid modulates the response of immune cells against malignant plasma cells: (i) attenuates the activation of dendritic cells and T effector cells, and (ii) inhibits monocytes migration and favors macrophage polarization towards the M2-phenotype (Figure 3). In conclusion, and similarly to other tumor milieu, the MM niche is a highly acidic and hypoxic site, where malignant plasma cells, bone and immune cells undergo a metabolic adaptation, leading toward immune tolerance [43].

The cells constituting the BM niche (osteoblasts, osteoclasts, stromal cells, endothelial and neoplastic cells, the most prominent ones) are floating in the surrounding available plasma. These are the main features making up a model where (i) to study a tumor in a closed site (e.g., MM in the BM niche), and (ii) to analyze minute amounts of plasma from the sample obtained for medical reasons (diagnostic or monitoring), and therefore, defining the immunochemical context of the working hypothesis, developed as follows. 

**Figure 4:**
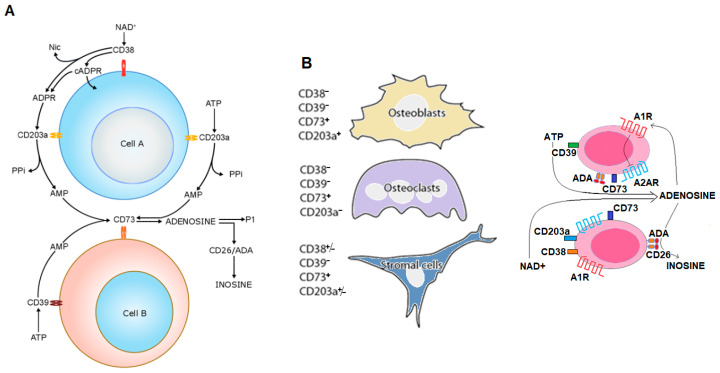
Schematic representation of discontinuous pathways of adenosine and inosine production. (**A**) NAD+ and ATP are metabolized to adenosine and inosine by the ectoenzymes belonging to the canonical (Cell B) and non-canonical (Cell A) pathways of nucleoside production. Adenosine can directly bind to purinergic receptors or be processed and transformed into inosine by CD26/ADA [47]. (**B**) Conceptual model of the role of ADO production, metabolism and receptor ligation in the malignant MM cells self-safeguarding. MM cells prevent the induction of an autocrine immunosuppression by abrogating A2AR-mediated signaling (by A1R activation) and deamination of ADO catalyzed by the CD26/ADA complex. Consequently, this versatile strategy would provide a safety lock of MM cells.

Our earlier studies on the biochemical background of MM indicate that ADO can be released from malignant cells, or produced from extracellular nucleotides of adenine (e.g., ATP and NAD^+^/NADP^+^) possibly leaking from damaged cells or actively released by connexin hemichannels during MM progression. These nucleotides are metabolized by ectoenzymes from different cells to produce locally and, bona fide in blood, increasing concentrations of ADO (and INO). Through this mechanism, adenosinergic pathways contribute to (i) malignant plasma cell preservation, as well as (ii) chastening of metabolic homeostasis to down-regulate anti-tumor immunosurveillance. Additionally, this in effect results in malignant plasma cell escape from immune killing (Figure 4A,B).

The expression and function of the NAD^+^/ATP dependent-adenosinergic pathways are up- or down-modulated under normoxic or hypoxic conditions, the latter prevalently found in closed acidic tumor environments. The metabolic activity associated with CD38 starts with the initial disassembly of NAD^+^/NADP^+^ and ends with the production of ADO (adenosinergic activity), provided that CD38 is operating in the presence of other ectoenzymes. The BM niche contains cells expressing different ectoenzymes (one of the richest conglomerates of such molecules): out of these, relevant is ectonucleotide pyrophosphatase/phosphodiesterase-1 (ENPP1), a molecule also known as plasma cell-1 (PC-1), the first marker identified on MM cells (now CD203a) [48]. Locally, the tandem CD38/CD203a, chained to the ectonucleotidase (eNT) CD73, composes a pathway for extracellular production of immunosuppressive ADO, as shown in Figure 4A. The metabolic conditions created in the BM by MM malignant cells make it possible to bypass the canonical adenosinergic pathway based on the conversion of ATP to AMP by the ecto-nucleoside triphosphate diphosphohydrolase (ENTPD-1) CD39 [49]. Therefore, it can be concluded that CD38 is the dominant enzyme for ADO generation in the MM environment, whereas the CD39/CD73 axis is compromised in the hypoxic acidic pH of the MM niche (Figure 3). ADO produced extracellularly may be metabolized to INO by the soluble adenosine deaminase (ADA) anchored to the plasma membrane of CD26 and can also modulate cell metabolism through ligation of one of four (A1, A2A, A2B, and A3) P1-adenosinergic receptors (ADORs), expressed by normal immune and non-immune cells as well as by MM cells [50].

ADO effects are associated with immunosuppression either in hematological or solid tumors (and normal immune cells). Indeed, evidence supporting the described mechanisms were confirmed in different normal cells (e.g., T- and B-, NK-, MDSC, among others) as well as in cells hijacked by pathological processes (T-, plasma-, leukemic-, melanoma-, glioblastoma- and non-small lung cancer-cells) [49,51,52,53,54]. In detail, CD56^bright^CD16^+^ NK cells produce ADO through a CD38-mediated pathway. The functional relevance is that these NK cells operate as activated immune effector cells by inhibiting autologous CD4^+^ T cell proliferation [55]. The immune escape strategy developed by MM cells is shared also by melanoma cells, where ADO suppresses CD4^+^ T cell proliferation, with immune suppression and loss of immune surveillance [56].

The hypothesis is that ADO produced in the BM niche may contribute to the general anergy observed in MM patients. Proof-of-concept supporting this view, was derived by linking the ADO levels of the niche with the clinical outcome of MM patients. The results demonstrated (i) that plasma levels of ADO are high in MM patients and (ii) such quantitative values increase with disease progression. Indeed, ADO levels in patients with symptomatic MM reflected the International Staging System (ISS) classification. A practical consequence of these findings is that ADO levels could potentially be used to monitor disease progression. An analysis on a larger number of patients may indeed validate the hypothesis that ADO levels could be predictive of disease outcome [57]. Notwithstanding, recent experimental data on ADO levels in MM obtained by mass spectroscopy support our conclusions [58].

## 6. CD38 in the Polarization and Release of Extracellular Vesicles (EV)

As ADO’s half-life in vivo is very short, we considered the possibility that MM cells were able to deliver a variety of signals at a distance from the BM, via the release of extracellular vesicles (EV).

An approach was suggested by the observation that the interaction between a therapeutic antibody and the target molecule is followed by a marked reshaping of the membrane and by a significant modulation of the cytoskeleton [48]. Once CD38 is targeted by anti-CD38 mAbs, the antigen expression can be either internalized or externalized. Using confocal microscopy, DARA treatment yields a clustering of the CD38 molecules into polar aggregates, successively released as EV.

The hypothesis (at least in part) is that the CD38/antibody complexes are not internalized: here the affinity of the antibody or its presentation (bound by FcR^+^-cells) may play a role. The resulting extracellular EVs contain CD38 and other accompanying ectoenzymes as well as the therapeutic antibody. Deriving from cell membranes, the EV may leave the site of origin and reach biological fluids. Being covered by the therapeutic antibody, their fate is inevitably to be captured and internalized by cells expressing FcR. The internalization is certainly followed by functional modifications in the recipient cells [59].

## 7. Clinical Applications outside MM

The applications of therapeutic anti-CD38 antibodies are not limited to MM. Being the first anti-CD38 antibody approved, most of the available data concern DARA, however, second-generation anti-CD38 mAbs in development are being studied in other (non-MM) pathologic conditions.

In hematology, different studies have shown that DARA may enhance macrophage-mediated phagocytosis of Burkitt’s lymphoma cells in vitro [60] and further enhance lysis by ADCC (Antibody-dependent cellular cytotoxicity) and ADCP (Antibody-dependent cellular phagocytosis) of CLL (Chronic lymphocytic leukemia) cells both in vitro and in vivo [61,62,63]. Moreover, in ex vivo peripheral blood cells from CLL patients (and in a CLL patient-derived xenograft model in vivo), DARA was noted to deplete the B-regulatory and T-regulatory cell fractions but promoted downstream activation and expansion of PD-1^low^CD8^+^ cytotoxic T-cells. This observation teases a tantalizing angle by which anti-CD38 mAbs may influence the restoration of host anti-tumor immune response [64]. These preclinical studies laid the rationale for clinical testing of DARA in relapsed/refractory CLL (NCT04230304).

The activity of DARA has been noted in the treatment of post-transplant autoimmune hemolytic anemia, mostly in pediatric patients [65,66,67]. In such setting, DARA showed encouraging results also as a rescue therapy in pre-treated patients refractory to common drugs such as rituximab, alemtuzumab, bortezomib, mycophenolate mofetil (MMF), sirolimus, and ibrutinib [68]. DARA was also used as a treatment in more complex scenarios, such as Evans’ Syndrome [69].

Acute myeloid leukemia (AML) is another disease where the anti-tumor effects of DARA have been investigated. In combination with *all-trans* retinoic acid (ATRA), DARA enhanced cytotoxicity of AML cells [70], while as a single agent, DARA induced an inhibition of mitochondrial transfer from mesenchymal stromal cells to AML cells, thus reducing the tumor burden [71]. Moreover, the efficacy of anti-CD38 therapy was assessed also in post-transplant refractory B-acute lymphoblastic leukemia [72,73,74].

In virtue of the expression of CD38 by NK cells, a case report highlighted the effectiveness of DARA in the treatment of an aggressive extranodal NK cell lymphoma [75]. DARA was also tested in in vitro and in vivo models of Waldenström Macroglobulinemia, showing ADCC, ADCP, CDC (Complement-dependent cytotoxicity) and induction of direct apoptosis in ibrutinib-resistant cells [63,76]. The activity of DARA in combination with ibrutinib is currently under clinical investigation in patients with Waldenström Macroglobulinemia (NCT03679624).

Anti-CD38 antibody therapy was also evaluated in patients with amyloidosis. A multicentric retrospective study showed the feasibility and effectiveness of DARA in relapsed/refractory AL (Amyloid light chain) amyloidosis suggesting its use early in the course of disease [77]. Other supporting data can be found in the literature [78,79,80].

One very recent application of DARA comes from the field of radiology and nuclear medicine: a ^64^Cu-conjugated form of DARA was used in a non-invasive in vivo tracing method to highlight CD38^+^ hepatocellular carcinoma lesions in a murine model [81].

As a whole, the picture from basic and clinical (with correlative) analyses conducted to date undoubtedly demonstrates that targeting CD38 via therapeutic antibodies is a promising therapeutic option in several fields of medicine.

## 8. Self-Vaccination

A question from basic science is whether anti-MM antibody treatment would lead to the generation of novel endogenous tumor-specific antibodies along with T cell responses. Indeed, Atanackovich et al. reported that treatment with ISA induced, in a subgroup of MM patients, the ability to respond to MM antigens and also to selected domains of the CD38 molecule [82]. Importantly, this study suggested that patients who developed antigen (CD38 and tumor)-specific CD4^+^ and CD8^+^ T-cell responses, were those who responded to ISA therapy [82]. Similar experiments are ongoing with DARA (A.C. Faini, 2020, unpublished; A. Paulus, 2020, unpublished).

## 9. Conclusions

The conclusion of this review is that targeting CD38 via therapeutic antibodies is an innovative therapeutic approach in several fields of medicine, many of which are in nascent stages of investigation. These conclusions are inferred from in vivo investigations on CD38 biology and its applications; exploiting continuous cross-talk between basic and clinical science. 

The results obtained from these studies did not simply provide a proof-of-principle for the clinical validity of the approach but provoked a myriad of intriguing downstream investigations. On the target side, the results confirmed many of the findings observed several years ago in basic science, but also confirmed that CD38 is not a pure “T cell activation marker, but a highly complex molecule”. On the antibody side, a wide body of basic findings were reverbed on the therapeutic antibodies, now prevalently seen as immunotherapeutic agents.

However, there are still multiple open questions concerning the actual effect of anti-CD38 antibodies in vivo that remain to be amswered.

Of clinical relevance:

(1) Studies on the effects of therapeutic mAbs on CD38 enzymatic activities should deal with the analysis on physiological consequences aimed at understanding whether modulation of the cyclase/hydrolase enzymatic activities (which are <2% and >90%, respectively, of the total CD38 enzymatic activity) alter the receptorial/signaling properties of CD38 [6].

(2) Does the block of the enzymatic activities of CD38 influence the clinical outcome of MM patients and if so, what are the molecular mechanisms involved? The question remains puzzling and should be supported by results coming from basic research followed by clinical applications. This will be facilitated by the availability of ISA, the second therapeutic anti-CD38 mAb and whose action on CD38 enzymatic activity is known.

(3) With the expanding availability and clinical use of DARA, the emergence of DARA-refractory disease is being increasingly recognized. Considering the accessibility of ISA, which has recently been approved for therapy and features different functions, could this next-generation anti-CD38 mAb be considered for retreatment of DARA-refractory patients?

(4) Contemplating the dynamics of membrane interactions by the therapeutic antibodies, is it reasonable to hypothesize that the generation of antigen/antibody complexes may induce an in-situ vaccination effect in MM patients?

(5) Considering the multiple functions of CD38 and its prevalent expression by plasma cells, may one hypothesize its application as a therapeutic tool in the wide range of diseases caused by auto-antibodies? Initial data have already been published on Systemic Lupus Erytematosus (SLE) [83,84,85,86].

## References

[1] Köhler G., Milstein C. (1975). Continuous cultures of fused cells secreting antibody of predefined specificity. Nat. Cell Biol..

[2] Zhang Y., Iii R.O.W., Tucker H. (2020). Formulation strategies in immunotherapeutic pharmaceutical products. World J. Clin. Oncol..

[3] De Winde C.M., Elfrink S., Van Spriel A.B. (2017). Novel insights into membrane targeting of B cell lymphoma. Trends Cancer.

[4] Lanza F., Maffini E., Rondoni M., Massari E., Faini A.C., Malavasi F. (2020). CD22 Expression in B-Cell acute lymphoblastic leukemia: Biological significance and implications for inotuzumab therapy in adults. Cancers.

[5] Giuliani N., Malavasi F. (2019). Editorial: Immunotherapy in multiple myeloma. Front. Immunol..

[6] Malavasi F., Deaglio S., Funaro A., Ferrero E., Horenstein A.L., Ortolan E., Vaisitti T., Aydin S. (2008). Evolution and function of the ADP ribosyl cyclase/CD38 gene family in physiology and pathology. Physiol. Rev..

[7] Drach J., Zhao S., Malavasi F., Mehta K. (1993). Rapid induction of CD38 antigen on myeloid leukemia cells by all trans-retinoic acid. Biochem. Biophys. Res. Commun..

[8] Drach J., McQueen T., Engel H., Andreeff M., Robertson K.A., Collins S.J., Malavasi F., Mehta K. (1994). Retinoic acid-induced expression of CD38 antigen in myeloid cells is mediated through retinoic acid receptor-alpha. Cancer Res..

[9] Malavasi F. (2011). Editorial: CD38 and retinoids: A step toward a cure. J. Leukoc. Biol..

[10] Ferrero E., Faini A.C., Malavasi F. (2019). A phylogenetic view of the leukocyte ectonucleotidases. Immunol. Lett..

[11] Terhorst C., Van Agthoven A., LeClair K., Snow P., Reinherz E., Schlossman S. (1981). Biochemical studies of the human thymocyte cell-surface antigens T6, T9 and T10. Cell.

[12] Alessio M., Roggero S., Funaro A., De Monte L.B., Peruzzi L., Geuna M., Malavasi F. (1990). CD38 molecule: Structural and biochemical analysis on human T lymphocytes, thymocytes, and plasma cells. J. Immunol..

[13] Hara-Yokoyama M., Kukimoto-Niino M., Terasawa K., Harumiya S., Podyma-Inoue K.A., Hino N., Sakamoto K., Itoh S., Hashii N., Hiruta Y. (2012). Tetrameric interaction of the ectoenzyme CD38 on the cell surface enables its catalytic and raft-association activities. Structure.

[14] Liu Q., Kriksunov I.A., Graeff R., Munshi C., Hao Q., Hao Q. (2005). Crystal structure of human CD38 extracellular domain. Structure.

[15] Funaro A., Horenstein A.L., Calosso L., Morra M., Tarocco R.P., Franco L., De Flora A., Malavasi F. (1996). Identification and characterization of an active soluble form of human CD38 in normal and pathological fluids. Int. Immunol..

[16] Horenstein L.A., Stockinger H., Imhof A.B., Malavasi F. (1998). CD38 binding to human myeloid cells is mediated by mouse and human CD31. Biochem. J..

[17] Deaglio S., Dianzani U., Horenstein A.L., Fernández J.E., Van Kooten C., Bragardo M., Funaro A., Garbarino G., Di Virgilio F., Banchereau J. (1996). Human CD38 ligand. A 120-KDA protein predominantly expressed on endothelial cells. J. Immunol..

[18] Deaglio S., Morra M., Mallone R., Ausiello C.M., Prager E., Garbarino G., Dianzani U., Stockinger H., Malavasi F. (1998). Human CD38 (ADP-ribosyl cyclase) is a counter-receptor of CD31, an Ig superfamily member. J. Immunol..

[19] Deaglio S., Mallone R., Baj G., Donati D., Giraudo G., Corno F., Bruzzone S., Geuna M., Ausiello C., Malavasi F. (2001). Human CD38 and its ligand CD31 define a unique lamina propria T lymphocyte signaling pathway. FASEB J..

[20] Hu Y., Liu H., Fang C., Li C., Xhyliu F., Dysert H., Bodo J., Habermehl G., Russell B.E., Li W. (2020). Targeting of CD38 by the tumor suppressor miR-26a serves as a novel potential therapeutic agent in multiple myeloma. Cancer Res..

[21] Zubiaur M., Izquierdo M., Terhorst C., Malavasi F., Sancho J. (1997). CD38 ligation results in activation of the Raf-1/mitogen-activated protein kinase and the CD3-zeta/zeta-associated protein-70 signaling pathways in Jurkat T lymphocytes. J. Immunol..

[22] Morra M., Zubiaur M., Terhorst C., Sancho J., Malavasi F. (1998). CD38 is functionally dependent on the TCR/CD3 complex in human T cells. FASEB J..

[23] Deaglio S., Capobianco A., Bergui L., Dürig J., Morabito F., Dührsen U., Malavasi F. (2003). CD38 is a signaling molecule in B-cell chronic lymphocytic leukemia cells. Blood.

[24] Deaglio S., Zubiaur M., Gregorini A., Bottarel F., Ausiello C.M., Dianzani U., Sancho J., Malavasi F. (2002). Human CD38 and CD16 are functionally dependent and physically associated in natural killer cells. Blood.

[25] Mallone R., Funaro A., Zubiaur M., Baj G., Ausiello C.M., Tacchetti C., Sancho J., Grossi C., Malavasi F. (2001). Signaling through CD38 induces NK cell activation. Int. Immunol..

[26] Zilber M.-T., Gregory S., Mallone R., Deaglio S., Malavasi F., Charron M., Gelin C. (2000). CD38 expressed on human monocytes: A coaccessory molecule in the superantigen-induced proliferation. Proc. Natl. Acad. Sci. USA.

[27] Lee H.C., Aarhus R. (1991). ADP-ribosyl cyclase: An enzyme that cyclizes NAD+ into a calcium-mobilizing metabolite. Cell Regul..

[28] Idzko M., Ferrari D., Eltzschig H.K. (2014). Nucleotide signalling during inflammation. Nat. Cell Biol..

[29] Deng Q.W., Zhang J., Li T., He W.M., Fang L., Lee H.C., Zhao Y.J. (2019). The transferrin receptor CD71 regulates type II CD38, revealing tight topological compartmentalization of intracellular cyclic ADP-ribose production. J. Biol. Chem..

[30] Lee H.C., Zhao Y.J. (2019). Resolving the topological enigma in Ca^2+^ signaling by cyclic ADP-ribose and NAADP. J. Biol. Chem..

[31] Lund F.E. (2006). Signaling properties of CD38 in the mouse immune system: Enzyme-dependent and -independent roles in immunity. Mol. Med..

[32] Jin D., Liu H.-X., Hirai H., Torashima T., Nagai T., Lopatina O., Shnayder N.A., Yamada K., Noda M., Seike T. (2007). CD38 is critical for social behaviour by regulating oxytocin secretion. Nat. Cell Biol..

[33] Chini E.N. (2009). CD38 as a regulator of cellular NAD: A novel potential pharmacological target for metabolic conditions. Curr. Pharm. Des..

[34] Deaglio S., Vaisitti T., Aydin S., Ferrero E., Malavasi F. (2006). In-tandem insight from basic science combined with clinical research: CD38 as both marker and key component of the pathogenetic network underlying chronic lymphocytic leukemia. Blood.

[35] Martin T.G., Corzo K., Chiron M., Van De Velde H., Abbadessa G., Campana F., Solanki M., Meng R., Lee H., Wiederschain D. (2019). Therapeutic opportunities with pharmacological inhibition of CD38 with isatuximab. Cells.

[36] Van De Donk N.W.C.J., Richardson P.G., Malavasi F. (2018). CD38 antibodies in multiple myeloma: Back to the future. Blood.

[37] An G., Jiang M.H., Acharya C., Zhong B.M.Y., Cai T., Yang G., Song Z., Theilhaber J., Adrian F., Tai Y.-T. (2014). SAR 650984, a therapeutic anti-CD38 monoclonal antibody, blocks CD38-CD31 interaction in multiple myeloma. Blood.

[38] Moreno L., Perez C., Zabaleta A., Manrique I., Alignani D., Ajona D., Blanco L., Lasa M., Maiso P., Rodriguez I. (2019). The mechanism of action of the anti-CD38 monoclonal antibody Isatuximab in multiple myeloma. Clin. Cancer Res..

[39] Malavasi F., Faini A.C. (2019). Mechanism of action of a new anti-CD38 antibody: Enhancing myeloma immunotherapy. Clin. Cancer Res..

[40] Krejcik J., Casneuf T., Nijhof I.S., Verbist B., Bald J., Plesner T., Syed K., Liu K., Van De Donk N.W.C.J., Weiss B.M. (2016). Daratumumab depletes CD38+ immune regulatory cells, promotes T-cell expansion, and skews T-cell repertoire in multiple myeloma. Blood.

[41] Horenstein A.L., Chillemi A., Quarona V., Zito A., Mariani V., Faini A.C., Morandi F., Schiavoni I., Ausiello C.M., Malavasi F. (2017). Antibody mimicry, receptors and clinical applications. Hum. Antibodies.

[42] Hogan K.A., Chini C.C.S., Chini E.N. (2019). The multi-faceted ecto-enzyme CD38: Roles in immunomodulation, cancer, aging, and metabolic diseases. Front. Immunol..

[43] Horenstein A.L., Bracci C., Morandi F., Malavasi F. (2019). CD38 in adenosinergic pathways and metabolic re-programming in human multiple myeloma cells: In-tandem insights from basic science to therapy. Front. Immunol..

[44] Van De Donk N.W.C.J. (2018). Immunomodulatory effects of CD38-targeting antibodies. Immunol. Lett..

[45] Yagui K., Shimada F., Mimura M., Hashimoto N., Suzuki Y., Tokuyama Y., Nata K., Tohgo A., Ikehata F., Takasawa S. (1998). A missense mutation in the CD38 gene, a novel factor for insulin secretion: Association with Type II diabetes mellitus in Japanese subjects and evidence of abnormal function when expressed in vitro. Diabetologia.

[46] Pham A., Mahindra A. (2019). Solitary plasmacytoma: A review of diagnosis and management. Curr. Hematol. Malign. Rep..

[47] Morandi F., Marimpietri D., Horenstein A.L., Bolzoni M., Toscani D., Costa F., Castella B., Faini A.C., Massaia M., Pistoia V. (2018). Microvesicles released from multiple myeloma cells are equipped with ectoenzymes belonging to canonical and non-canonical adenosinergic pathways and produce adenosine from ATP and NAD+. OncoImmunology.

[48] Belli S.I., Goding J.W. (1994). Biochemical characterization of human PC-1, an enzyme possessing alkaline phosphodiesterase I and nucleotide pyrophosphatase activities. JBIC J. Biol. Inorg. Chem..

[49] Horenstein A.L., Chillemi A., Zaccarello G., Bruzzone S., Quarona V., Zito A., Serra S., Malavasi F. (2013). A CD38/CD203a/CD73 ectoenzymatic pathway independent of CD39 drives a novel adenosinergic loop in human T lymphocytes. OncoImmunology.

[50] Horenstein A.L., Chillemi A., Quarona V., Zito A., Roato I., Morandi F., Marimpietri D., Bolzoni M., Toscani D., Oldham R.J. (2015). NAD+-metabolizing ectoenzymes in remodeling tumor–host interactions: The human myeloma model. Cells.

[51] Quarona V., Ferri V., Chillemi A., Bolzoni M., Mancini C., Zaccarello G., Roato I., Morandi F., Marimpietri D., Faccani G. (2015). Unraveling the contribution of ectoenzymes to myeloma life and survival in the bone marrow niche: Ectoenzymes and the myeloma niche. Ann. N. Y. Acad. Sci..

[52] Horenstein A.L., Morandi F., Bracci C., Pistoia V., Malavasi F. (2019). Functional insights into nucleotide-metabolizing ectoenzymes expressed by bone marrow-resident cells in patients with multiple myeloma. Immunol. Lett..

[53] Ferretti E., Horenstein A.L., Canzonetta C., Costa F., Morandi F. (2018). Canonical and non-canonical adenosinergic pathways. Immunol. Lett..

[54] Horenstein A.L., Chillemi A., Zini R., Quarona V., Bianchi N., Manfredini R., Gambari R., Malavasi F., Ferrari D. (2018). Cytokine-induced killer cells express CD39, CD38, CD203a, CD73 Ectoenzymes and P1 adenosinergic receptors. Front. Pharmacol..

[55] Morandi F., Horenstein A.L., Chillemi A., Quarona V., Chiesa S., Imperatori A., Zanellato S., Mortara L., Gattorno M., Pistoia V. (2015). CD56brightCD16−NK cells produce adenosine through a CD38-mediated pathway and act as regulatory cells inhibiting autologous CD4+T cell proliferation. J. Immunol..

[56] Morandi F., Morandi B., Horenstein A.L., Chillemi A., Quarona V., Zaccarello G., Carrega P., Ferlazzo G., Mingari M.C., Moretta L. (2015). A non-canonical adenosinergic pathway led by CD38 in human melanoma cells induces suppression of T cell proliferation. Oncotarget.

[57] Horenstein A.L., Quarona V., Toscani D., Costa F., Chillemi A., Pistoia V., Giuliani N., Malavasi F. (2016). Adenosine generated in the bone marrow niche through a CD38-mediated pathway correlates with progression of human myeloma. Mol. Med..

[58] Yang R., Elsaadi S., Misund K., Abdollahi P., Vandsemb E.N., Moen S.H., Kusnierczyk A., Slupphaug G., Standal T., Waage A. (2020). Conversion of ATP to adenosine by CD39 and CD73 in multiple myeloma can be successfully targeted together with adenosine receptor A2A blockade. J. Immunother. Cancer.

[59] Chillemi A., Quarona V., Zito A., Morandi F., Marimpietri D., Cuccioloni M., Robert O.J., Mark C.S., Bolzoni M., Toscani D. (2015). Generation and characterization of microvesicles after daratumumab interaction with myeloma cells. Blood.

[60] Overdijk M.B., Verploegen S., Bögels M., Van Egmond M., Van Bueren J.J.L., Mutis T., Groen R.W.J., Breij E., Martens A.C.M., Bleeker W.K. (2015). Antibody-mediated phagocytosis contributes to the anti-tumor activity of the therapeutic antibody daratumumab in lymphoma and multiple myeloma. mAbs.

[61] Matas-Céspedes A., Vidal-Crespo A., Rodriguez V., Villamor N., Delgado J., Giné E., Roca-Ho H., Menéndez P., Campo E., López-Guillermo A. (2016). The human CD38 monoclonal antibody daratumumab shows antitumor activity and hampers leukemia–microenvironment interactions in chronic lymphocytic leukemia. Clin. Cancer Res..

[62] Manna A., Aulakh S., Jani P., Ahmed S., Akhtar S., Coignet M., Heckman M.G., Meghji Z., Bhatia K., Sharma A. (2019). Targeting CD38 enhances the antileukemic activity of ibrutinib in chronic lymphocytic leukemia. Clin. Cancer Res..

[63] Paulus A., Manna A., Akhtar S., Paulus S.M., Sharma M., Coignet M.V., Jiang L., Roy V., Witzig T.E., Ansell S.M. (2018). Targeting CD38 with daratumumab is lethal to Waldenström macroglobulinaemia cells. Br. J. Haematol..

[64] Manna A., Kellett T., Aulakh S., Lewis-Tuffin L.J., Dutta N., Knutson K., Chini E., Pinilla-Ibarz J., Lamanna N., Manochakian R. (2020). Targeting CD38 is lethal to Breg-like chronic lymphocytic leukemia cells and Tregs, but restores CD8+ T-cell responses. Blood Adv..

[65] Tolbert V.P., Goldsby R., Huang J., Shimano K., Melton A., Willert J., Horn B.N., Dvorak C.C., Wahlstrom J.T. (2016). Daratumumab is effective in the treatment of refractory post-transplant autoimmune hemolytic anemia: A pediatric case report. Blood.

[66] Cooling L., Hugan S. (2019). Daratumumab in combination with standard treatment for autoimmune hemolytic anemia in a pediatric patient. Transfusion.

[67] Even-Or E., Eddin A.N., Shadur B., Schejter Y.D., Najajreh M., Zelig O., Zaidman I., Stepensky P. (2019). Successful treatment with daratumumab for post-HSCT refractory hemolytic anemia. Pediatr. Blood Cancer.

[68] Schuetz C., Hoenig M., Moshous D., Weinstock C., Castelle M., Bendavid M., Shimano K., Tolbert V., Schulz A.S., Dvorak C.C. (2018). Daratumumab in life-threatening autoimmune hemolytic anemia following hematopoietic stem cell transplantation. Blood Adv..

[69] Blennerhassett R., Sudini L., Gottlieb D., Bhattacharyya A. (2019). Post-allogeneic transplant Evans syndrome successfully treated with daratumumab. Br. J. Haematol..

[70] Buteyn N.J., Fatehchand K., Santhanam R., Fang H., Dettorre G.M., Gautam S., Harrington B., Henderson S.E., Merchand-Reyes G., Mo X. (2018). Anti-leukemic effects of all-trans retinoic acid in combination with Daratumumab in acute myeloid leukemia. Int. Immunol..

[71] Mistry J.J., Moore J.A., Kumar P., Marlein C.R., Hellmich C., Pillinger G., Jibril A., Di Palma F., Collins A., Bowles K.M. (2020). Daratumumab inhibits acute myeloid leukaemia metabolic capacity by blocking mitochondrial transfer from mesenchymal stromal cells. Haematologica.

[72] Zhang Y., Xue S., Liu F., Wang J. (2020). Daratumumab for quick and sustained remission in post-transplant relapsed/refractory acute lymphoblastic leukemia. Leuk. Res..

[73] Myers M.A., McPhail L.C., Snyderman R. (1985). Redistribution of protein kinase C activity in human monocytes: Correlation with activation of the respiratory burst. J. Immunol..

[74] Ofran Y., Ringelstein-Harlev S., Slouzkey I., Zuckerman T., Yehudai-Ofir D., Henig I., Beyar-Katz O., Hayun M., Frisch A. (2020). Daratumumab for eradication of minimal residual disease in high-risk advanced relapse of T-cell/CD19/CD22-negative acute lymphoblastic leukemia. Leukemia.

[75] Hari P., Raj R.V., Olteanu H. (2016). Targeting CD38 in refractory extranodal natural killer cell–T-cell lymphoma. N. Engl. J. Med..

[76] Paulus A., Akhtar S., Bashir Y., Paulus S.M., Yousaf H., Roy V., Ailawadhi S., Ansell S., Witzig T.E., Chanan-Khan A.A. (2016). Drug resistance alters CD38 expression and in vitro response to daratumumab in waldenstrom macroglobulinemia cells. Blood.

[77] Lecumberri R., Krsnik I., Askari E., Sirvent M., González-Pérez M.S., Escalante F., Pradillo V., Tamariz L.E., Cánovas V., Alegre A. (2020). Treatment with daratumumab in patients with relapsed/refractory AL amyloidosis: A multicentric retrospective study and review of the literature. Amyloid.

[78] Canichella M., Serrao A., Annechini G., D’Elia G.M., De Luca M.L., Pulsoni A. (2018). Long-term response to daratumumab in a patient with advanced immunoglobulin light-chain (AL) amyloidosis with organ damage. Ann. Hematol..

[79] Deshpande S., Gertz M.A., Dispenzieri A., Kumar S.S., Parikh S.A., Muchtar E. (2020). daratumumab as successful initial therapy for AL amyloidosis with nerve involvement. Leuk. Lymphoma.

[80] Roccatello D., Fenoglio R., Sciascia S., Naretto C., Rossi D., Ferro M., Barreca A., Malavasi F., Baldovino S. (2020). CD38 and anti-CD38 monoclonal antibodies in AL amyloidosis: Targeting plasma cells and beyond. Int. J. Mol. Sci..

[81] Li S., England C.G., Ehlerding E.B., Kutyreff C.J., Engle J.W., Jiang D., Cai W. (2019). ImmunoPET imaging of CD38 expression in hepatocellular carcinoma using 64Cu-labeled daratumumab. Am. J. Transl. Res..

[82] Atanackovic D., Yousef S., Shorter C., Tantravahi S.K., Steinbach M., Iglesias F., Sborov D., Radhakrishnan S.V., Chiron M., Miles R. (2019). In vivo vaccination effect in multiple myeloma patients treated with the monoclonal antibody isatuximab. Leukemia.

[83] Katsuyama E., Suarez-Fueyo A., Bradley S.J., Mizui M., Marin A.V., Mulki L., Krishfield S., Malavasi F., Yoon J., Sui S.J.H. (2020). The CD38/NAD/SIRTUIN1/EZH2 axis mitigates cytotoxic CD8 T Cell function and identifies patients with SLE prone to infections. Cell Rep..

[84] Chakraborty P., Mehrotra S. (2020). CD38: Modulating histone methyltransferase EZH2 activity in SLE. Trends Immunol..

[85] Kumar S.U., Kumar D.T., Siva R., Doss C.G.P., Younes S., Younes N., Sidenna M., Zayed H. (2020). Dysregulation of signaling pathways due to differentially expressed genes from the B-Cell transcriptomes of systemic lupus erythematosus patients—A bioinformatics approach. Front. Bioeng. Biotechnol..

[86] Korver W., Carsillo M., Yuan J., Idamakanti N., Wagoner M., Shi P., Xia C.Q., Smithson G., McLean L., Zalevsky J. (2019). A Reduction in B, T, and natural killer cells expressing CD38 by TAK-079 inhibits the induction and progression of collagen-induced arthritis in cynomolgus monkeys. J. Pharmacol. Exp. Ther..

